# Calcium Carbonate–Carboxymethyl Chitosan Hybrid Materials

**DOI:** 10.3390/ma14123336

**Published:** 2021-06-16

**Authors:** Maria E. Fortună, Elena Ungureanu, Carmen D. Jitareanu

**Affiliations:** 1 Institute of Macromolecular Chemistry “Petru Poni”, 41A Grigore Ghica Voda Alley, 700487 Iasi, Romania; fortuna.maria@icmpp.ro; 2 Exact Sciences Department, Faculty of Horticulture, “Ion Ionescu de la Brad” University of Life Sciences Iasi, 3 Mihail Sadoveanu Alley, 700490 Iasi, Romania; 3 Plant Science Department, Faculty of Agriculture, “Ion Ionescu de la Brad” University of Life Sciences Iasi, 3 Mihail Sadoveanu Alley, 700490 Iasi, Romania; doinaj@uaiasi.ro

**Keywords:** calcium carbonate precipitated (PCC), chitosan (CH), carboxymethyl chitosan (CMC), hybrid materials, paper loading

## Abstract

In the present work, precipitated calcium carbonate (PCC) and carboxymethyl chitosan (CMC) were prepared to obtain new hybrid materials used in papermaking. In the first step, occurred the precipitation of CaCO_3_ in solution containing CMC at different levels (0.5%, 1%, and 1.5%). In the second step, PCC–CMC hybrid material (25%) was added to pulp suspension, and the sheets were made. The effect of PCC–CMC on paper properties (mechanical and optical) was systematically investigated. Breaking length, the brightness and opacity of the sheets obtained with the PCC–CMC material were better than the sheets fabricated with the unmodified PCC at similar levels of content.

## 1. Introduction

In the last years, numerous researches have been utilized to fabricate hybrid materials consisting of organic and inorganic components with special chemical and physical properties for the development of numerous promising products in different fields [1,2,3,4].

Some research in the field of papermaking has shown the compatibility of precipitated calcium carbonate (PCC) with organic compounds [5,6,7,8,9]. The most modifications of precipitated calcium carbonate (PCC) regarding the improvement of its filler properties are those related to the use of bio-additives (starch, alginate, cellulose, and chitosan) which reduce the negative impact of fillers on the paper properties and on the technological process [10,11,12,13]. Modification of the calcium carbonate particles with the chitosan can show good benefits of using fillers in papermaking due to reducing the cost price by the partial replacement of fibers and also improving the properties of paper [6,14]. Chitosan and its derivatives have been considered as versatile candidates for preparing specific inorganic–organic hybrid materials. However, the modification of chitosan in its derivatives is necessary to confer solubility and amphoteric character. Thus, a biocompatible and biodegradable derivative of chitosan is carboxymethyl chitosan which is soluble in water [15]. In the last decade, carboxymethyl chitosan materials have been received increasing attention due to the fact that it is a source of renewable and biodegradable material and, on the other hand, due to functionality through application in biology, technology, biotechnology, medicine and agriculture [16,17]. However, trends in the paper industry to use a higher amount of secondary fiber and fillers can give negative consequences for both the papermaking process and the paper’s properties. For example, a large amount of filler material introduced into the pulp paper (20–40%) will decrease the strength of the obtained paper, but this can be compensated by the use of retention additives. Retention is the most widely used parameter for an effective characteristic of additive or additive systems. By combining different additives, we can control some unwanted phenomena such as the decrease in the environmental impact of papermaking by lowering the process water loading as a result of high filler retention and by reducing the energy consumption for the pulp stock preparation and wastewater treatment [18].

The chitosan has already been evaluated as a filler material for the papermaking industry [19,20]. However, the influence of chitosan on structural changes in PCC and their effect on paper properties has not yet been reported in the literature. In this sense, in this work, to control the particle size of PCC and to obtain new hybrid materials with modified PCC, carboxymethyl chitosan was utilized. The preparation of PCC–CMC hybrid particles did not involve any organic solvent and could offer good control over the morphology of particles with relatively narrow size distributions. The soluble chitosan is synthesized under alkaline conditions by reaction with mono-chloroacetic acid. This additive was tested in papermaking and proved to be effective. Based on these results, in this work, PCC was obtained by the double exchange reaction in the presence of carboxymethyl chitosan (CMC).

## 2. Experimental

### 2.1. Materials

- Chitosan was purchased from Vanson, Inc. Company. The main characteristics of chitosan are acetylation degree—20.8%, molecular weight—415.000 g/mol, cationic charge—4500 μeq/g.

Carboxymethyl chitosan, soluble in water (CMC), was obtained from chitosan according to previous methods [21,22]. In total, 2 g of chitosan was dispersed in 50 mL isopropanol and stirred using a magnetic stirrer at room temperature for 2 h. Then, 80 mL of aqueous NaOH solution (60% *w/v*) and 100 mL of aqueous monochloroacetic acid solution (60% *w/v*) were added, and the mixture was heated with stirring at 65 °C for a further 8 h. The reaction mixture was then neutralized using HCl solution (4 M). After the removal of the residue by filtration, the resulting CMC was precipitated by adding methanol. The product was extensively washed with 80% ethanol and dried at room temperature. The substitution degree was 0.98.

- CaCl_2_ (96%) and Na_2_CO_3_ (99.8%) were used as chemicals for precipitated calcium carbonate—PCC.
- Softwood bleached kraft pulp refined at 30 °SR (Schopper-Riegler degree).

### 2.2. Experimental Procedure

In the following evaluations, calcium carbonate obtained from calcium chloride and sodium carbonate will be used to which were added variable amounts of carboxymethyl chitosan relative to PCC (0.5% CMC, 1% CMC, and 1.5% CMC).

PCC was obtained by the following reaction:

CaCl_2_ + Na_2_CO_3_ → 2NaCl + CaCO_3_ (1)

In a typical synthesis, CMC at different concentrations (0.5%, 1%, and 1.5%) was dissolved in deionized water, CaCl_2_ solution was added, and finally, the Na_2_CO_3_ was added slowly and was left at 25 °C for 10 h. Finally, the precipitated calcium carbonate crystals were filtered, then washed and dried at 45 °C.

**Figure 1:**
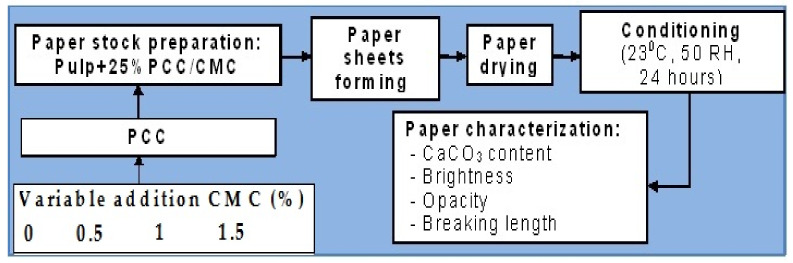
A schematic representation of the technological routes for the preparation of PCC–CMC and paper characterization.

Paper stock preparation was performed by adding precipitated calcium carbonate/carboxymethyl chitosan (PCC–CMC) into slurries of cellulosic fibers under stirring before the formation of paper; the dosage of calcium carbonate was established at 25% after several retention tests to obtaining paper hand sheets. After that, the pulp was washed in several steps for the complete removal of the free calcium carbonate particles (Figure 1).

### 2.3. Methods

The structures of unmodified chitosan (CH) and carboxymethyl chitosan (CMC) samples were studied by Fourier Transform Infra-Red (FTIR) spectroscopy, recorded using a Bruker Vertex 70 FTIR spectrometer.

The paper hand sheets were obtained on a Rapid-Köthen apparatus, at a standard basis weight of 70 g/m^2^; three series of hand sheets were prepared for each type of loaded pulp. Paper sheets were conditioned under standard conditions (24 h at 23 °C and 50% RH) and analyzed regarding the calcium carbonate content, according to the Tappi Standard—T413 (Tappi Test Methods, 1999). The calcium carbonate content is calculated from the ash content in the hand sheets, taking into account the incineration loss of the precipitated calcium carbonate. Calcium carbonate retention was determined on the Dynamic Drainage Jar (DDJ)device: from the paste with a consistency of 0.5%, homogenized, 500 mL are introduced into the vessel of the DDJ apparatus with the drain valve closed, while the stirring started; the paste is stirred for about 30 s, and then PCC or PCC–CMC are added according to the experimental programs. To collect the filtrate, stirring is stopped at the same time as opening the solenoid valve, about 30 mL of the filtrate is removed, and the next 100 mL are collected in a graduated vessel. The collected greasy water is filtered on quality filter paper, recalibrated, dried, and weighed. Retention of calcium carbonate was determined by calcination of the filter paper, at approximately 600 °C.

Opacity and brightness were determined according to ISO 2471, measured by the spectrophotometer L&W Elrepho 2000.

Sstrength properties: breaking length (Km) measured by Instron apparatus, according to ISO 1924 and burst factor (KPa.m^2^/g) measured by the Schopper-Dale apparatus, according to ISO 2758.

Particle size distribution of precipitated calcium carbonate was measured by a laser diffraction particle size analyzer (Sald-7001/Shimadzu Scientific Instrument).

X-ray diffraction patterns of calcium carbonate particulates were obtained on a D8 ADVANCE, Bruker-AXS apparatus, equipped with a transmission type goniometer, using nickel-filtered CuKα radiation (λ = 1.5418 Å) at 36 kV; the goniometer was scanned stepwise every 0.10° from 10 to 60° in the 2θ range. 

The morphology of PCC–CMC material and of the paper surface was visualized by scanning electron microscopy ((SEM) (VEGA/Tescan)).

## 3. Results and Discussion

### 3.1. CMC Modification

**Figure 2:**
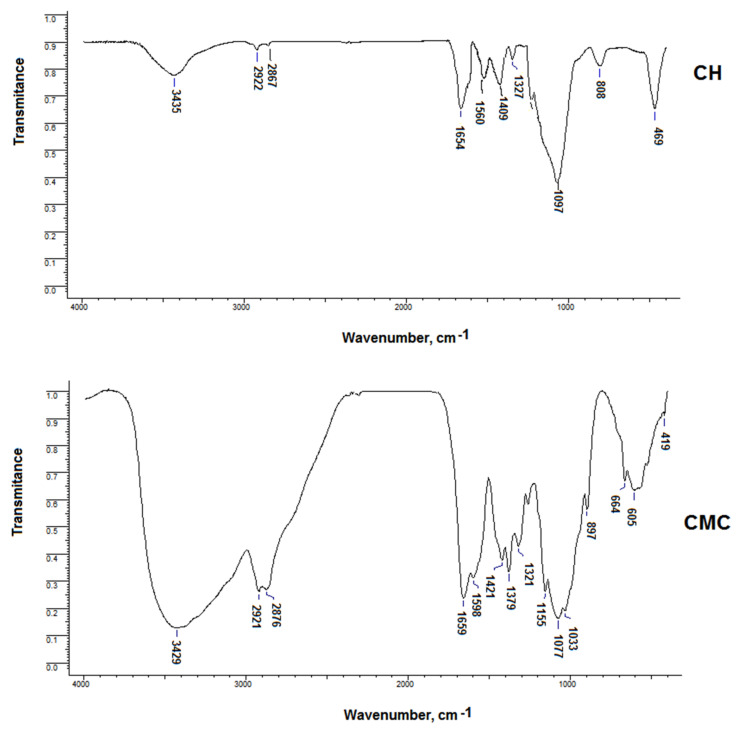
FTIR spectra of CH and CMC.

Both unmodified and modified carboxymethyl chitosan samples were characterized using FTIR spectroscopy (Figure 2). The modifications observed in the FTIR spectra appear due to the changes in the chemical structure.

According to FT-IR Spectra, as shown in Figure 2, the main characteristic peaks of chitosan are at 3435 (O-H and N-H), 2867–2922 (C-H stretch), 1560 (N-H bend), 1654 cm^−1^ (C=O stretch, amide I band), 1165 (bridge O stretch) and 1097 cm^−1^(C-O stretch).

For CMC, its spectrum is different from the spectrum of chitosan: 3429 (O-H and N-H bend); 2876 and 2921 (C-H stretch); 1598 (COO-); 1379 and 1421 (-CH_2-_ and -CH_3_-); 1077 and 1321 (C-O). CMC presents an absorption band at 1598 cm^−1^ specific for the COO- group, indicating that the carboxymethyl group is grafted onto the molecular chain of chitosan [23]. 

### 3.2. Surface Morphology 

**Figure 3:**
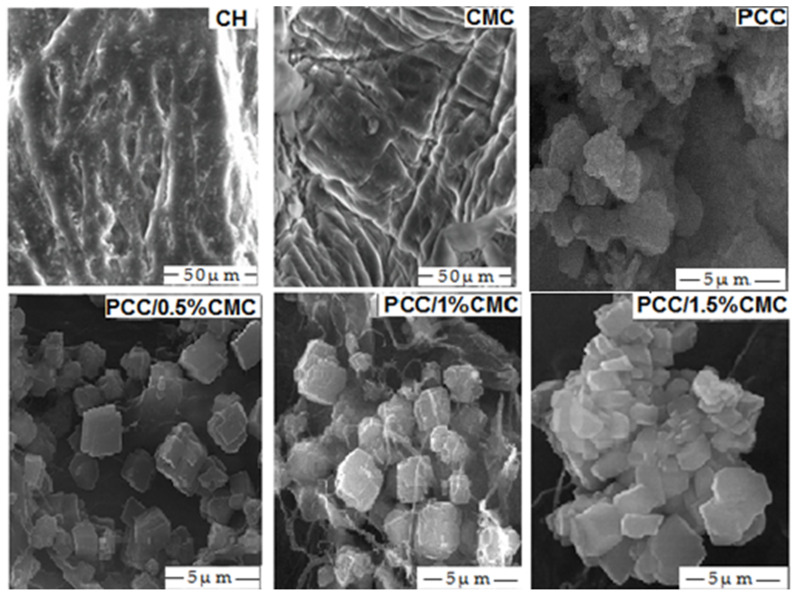
SEM images of CH, CMC, PCC, and PCC with addition CMC.

The morphology of CH, CMC, PCC, and PCC–CMC material hybrid was observed by Scanning Electron Microscope. The shape and dimensions of PCC particles, obtained in the laboratory at various CMC additions, were analyzed and the results obtained are presented in Figure 3.

The three polymorphic forms of precipitated calcium carbonate are calcite (thermodynamically stable at room temperature), vaterite, and aragonite, which are easier to be transformed into calcite. 

In the literature [4,24], following the reaction between CaCl_2_ + Na_2_CO_3_ at different temperatures, the calcite form was obtained at 25 °C. 

In our case, the examination of the SEM images (Figure 3) evidence that the forms are spindle-shaped and cubic type.

### 3.3. X-ray Analysis

For the synthesis of new filling materials with unique properties, it is very important to follow the particle size, polymorphism, and morphology. 

**Figure 4:**
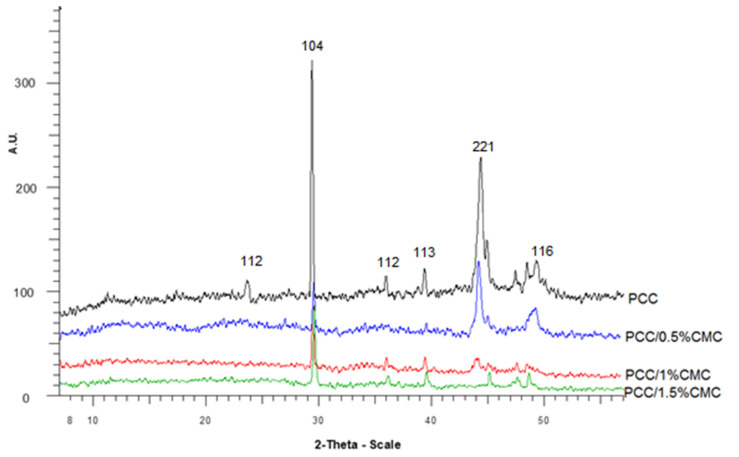
XRD patterns of PCC and PCC–CMC.

X-ray diffraction remains the main method for the identification of atom arrangement in minerals. In this study, X-ray analysis was applied to determine the polymorphic form of modified and unmodified calcium carbonate particles (Figure 4). 

XRD calibration graphs were constructed using the 104 reflections of calcite, the 221 reflections of aragonite, and the 110 reflections of vaterite.

According to with X-ray analysis, the peaks at 2θ of 23.5° (112) and 29.4° (104) are assigned with calcite, whereas 2θ of 36.1° (112), 44.8° (221), and 49.2° (116) are associated to the aragonite phase [25,26]. After introducing CMC in PCC dispersion, the intensity of peaks at 2θ = 23.5°, corresponding to the plane (112), and 2θ = 36.1°, corresponding to the plane (112), have disappeared. Additionally, the intensity of the peak at 29.4° associated to the plane (104) of calcite and 44.8° (221) of aragonite has been reduced. At a higher concentration of CMC (PCC/1.5% CMC), the peaks at 44.8° (221) and 49.2° (116) have disappeared.

PCC modified with CMC samples appeared as a mixture of the majority of calcite with smaller amounts of aragonite.

#### Particle Size of Modified and Unmodified PCC

**Figure 5:**
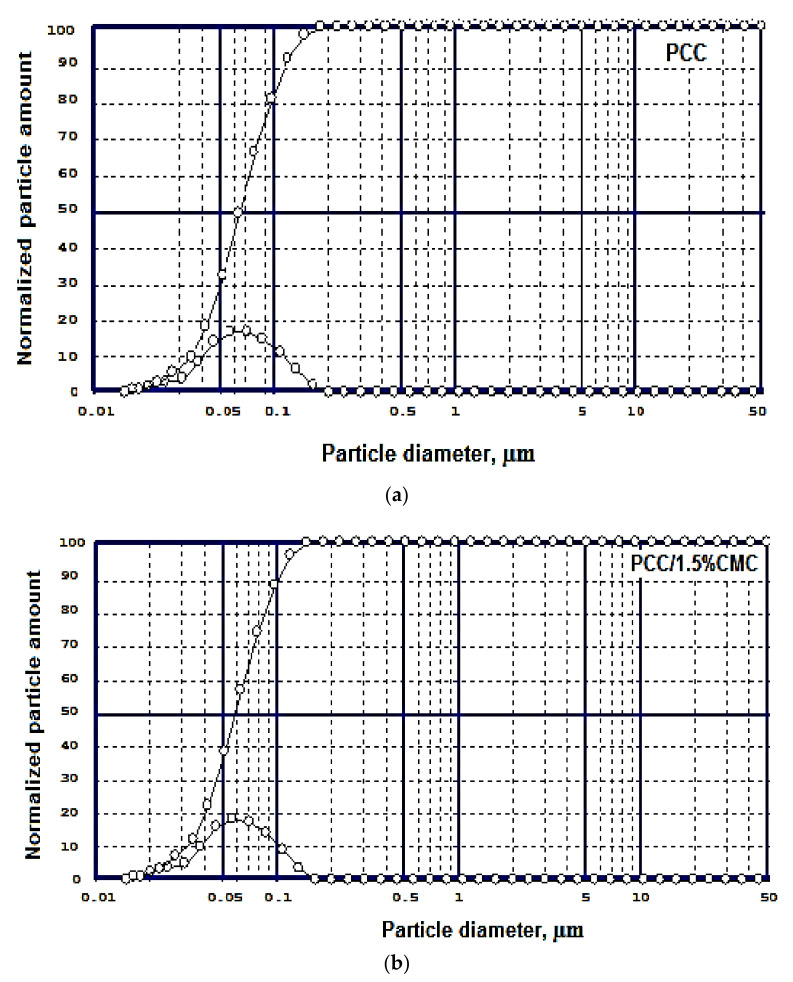
Particle size distribution of PCC (**a**) and PCC/1.5% CMC (**b**) (particle diameters and cumulative curve).

The size of unmodified PCC and CMC modified PCC were analyzed with a laser diffraction particle size analyzer (Figure 5). The PCC and PCC–CMC were submitted to ultrasound in distilled water for 10 min and analyzed.

It is found that with the increase in the carboxymethyl chitosan addition, the calcium carbonate particles obtained in the laboratory are slightly smaller than the control particles obtained without the addition of carboxymethyl chitosan, which should lead to better retention in the paper’s structure. 

From Figure 5, it was determined that PCC/1.5% CMC has the d_50_ of 0.058 μm, and particle distribution is into narrow diameters.

### 3.4. Properties of Filled Paper Sheets

#### 3.4.1. Retention

Current trends in the paper industry are to use a higher amount of secondary fiber, fillers and coating pigments have negative consequences for the paper properties. Thus, a large amount of filler material introduced into the pulp paper will decrease the strength of the obtained paper, but this can be compensated by the use of retention additives. Retention is the most widely used parameter for an effective characteristic of additive or additive systems.

Carboxymethyl chitosan can be used to improve the dry strength of the paper and to replace the cationic starch in the pulp composition in the manufacture of printing paper. 

**Figure 6:**
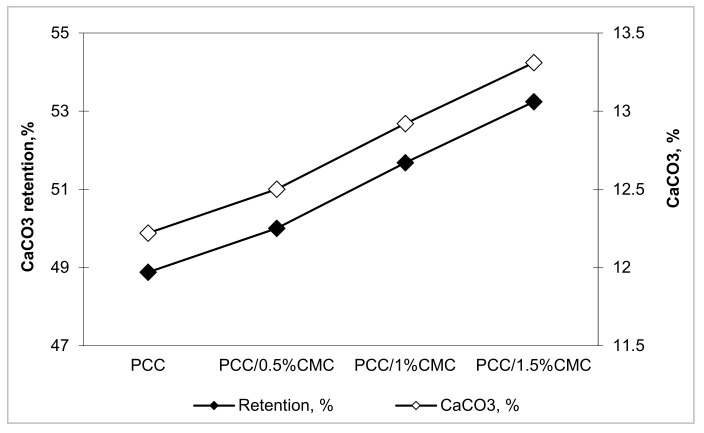
The influence of CMC addition on retention efficiency and calcium carbonate content in the paper.

Figure 6 shows the evolution of the retention yield and the CaCO_3_ amount in the sheets as a function of the addition of carboxymethyl chitosan in the pulp. The content of precipitated calcium carbonate without CMC is 12.2%, compared to 13.3% for PCC/1.5% CMC. Retention yield increases with increasing CaCO_3_ amount, which progressively increases with the CMC addition (from 48.9% for PCC without CMC to 53.2% for PCC/1.5% CMC). The use of CMC as an individual component leads to an aggregation mechanism that results in the formation of a compact structure, and consequently, retention and formation are advantageous.

#### 3.4.2. SEM Images of Paper Samples

**Figure 7:**
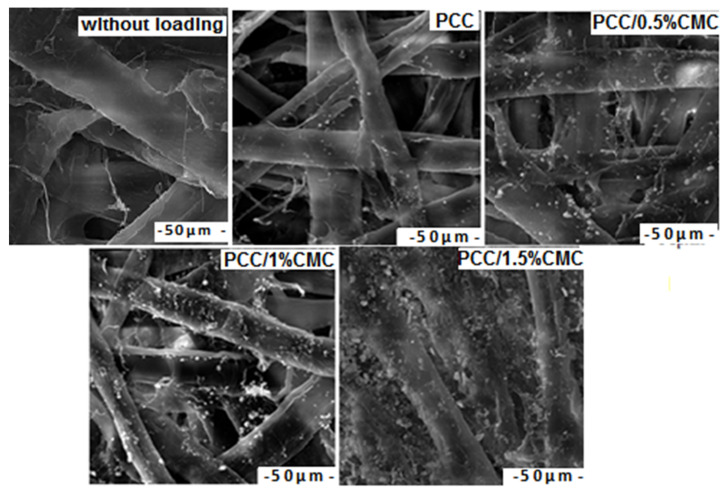
SEM micrographs for paper samples.

SEM images of paper samples with PCC and PCC–CMC are shown in Figure 7. As expected, the surface morphology of the samples with PCC–CMC was different from that with PCC, which confirms the importance of filler modification in structural change. Between fibers of pulp, some particles can be seen, and these are fulfilling the interfibers space, which leads to the improvement of physico-chemical properties. 

SEM analyses showed differences among the precipitated calcium carbonate, modified and unmodified with regard to particle size and distribution within the fibers, as reflected by the levels of the opacity and tensile strength of paper sheets. 

#### 3.4.3. Optical Properties

**Figure 8:**
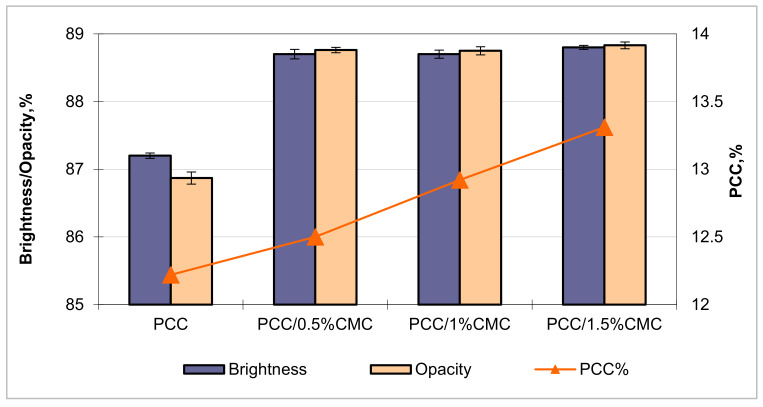
Brightness and opacity of paper samples.

Opacity is one of the properties of papers that depend on the content of the filler material and its distribution in the paper structure. The results in Figure 8 show the effects of CMC on the optical properties of the sheets. The brightness and opacity increase with the increase in the addition of CMC in the pulp. This can be attributed to the increase in the content of PCC between fibers and also due to the change in the sizes and distribution of particles.

#### 3.4.4. Mechanical Strength Properties

**Figure 9:**
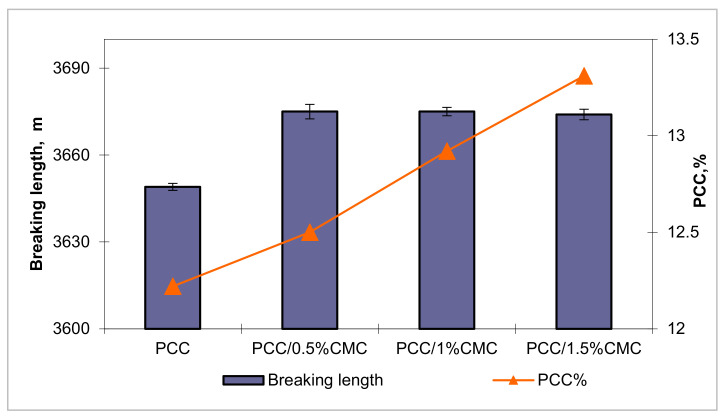
The effect of PCC modified with CMC of different levels on breaking length.

**Figure 10:**
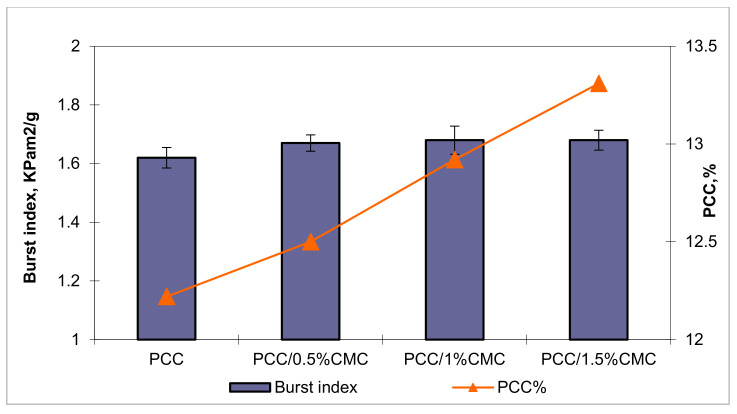
The effect of PCC modified with CMC of different addition levels on burst index.

The breaking length is significantly influenced by additives addition. The influence of filler in different levels of CMC on the mechanical properties of sheets is shown in Figure 9 and Figure 10.

For a correct evaluation of the efficiency of carboxymethyl chitosan on the strength of the paper, the values for the breaking length of the paper were calculated according to the calcium carbonate content. Figure 9 and Figure 10 highlight the positive effect of carboxymethyl chitosan on the strength properties. The increase in the breaking length for samples with the addition of PCC–CMC compared to those with PCC without CMC can be attributed to the formation of composite structures such as calcium carbonate–carboxymethyl chitosan.

This increase in paper strength for samples with the addition of PCC–CMC, compared to those with PCC obtained by the same method, in the absence of CMC, can also be explained by the fact that most fiber–pigment interfaces are made by the carboxymethyl chitosan film, which has the ability to develop hydrogen bonds.

Although the PCC content increases with the addition of CMC, the burst index is not affected, which implies that most of the calcium carbonate is precipitated in the pores of the fibers and fine material, the particles being much smaller. Under these conditions, the calcium carbonate particles being small do not contribute to the increase in interfibrillar spaces.

## 4. Conclusions

The particle size distribution of PCC modified with carboxymethyl chitosan was changed, but not too much, comparatively with PCC without CMC.

SEM images show that the calcium carbonate particles in the paper appear smaller by increasing the addition of CMC.

Retention of filler in dispersed form is also supported by a substantial increase in brightness and opacity.

The optical properties of sheets filled with PCC–CMC were improved in comparison to the PCC without CMC. On the other hand, sheets filled with PCC–CMC hybrid material reduced the loss of mechanical properties at the same PCC amount. 

## Data Availability

Not applicable.
